# Cloning of an Albino Mutation of *Arabidopsis thaliana* Using Mapping-by-Sequencing

**DOI:** 10.3390/ijms24044196

**Published:** 2023-02-20

**Authors:** Eva Rodríguez-Alcocer, Erundina Ruiz-Pérez, Ricardo Parreño, César Martínez-Guardiola, José Marcos Berna, Ayça Çakmak Pehlivanlı, Sara Jover-Gil, Héctor Candela

**Affiliations:** 1 Instituto de Bioingeniería, Universidad Miguel Hernández, Campus de Elche, 03202 Elche, Spain; 2 Department of Statistics, Mimar Sinan Fine Arts University, 34380 Istanbul, Turkey

**Keywords:** mapping-by-sequencing, Fisher’s exact test, chloroplast, HSP90 proteins, heat shock proteins, *Arabidopsis thaliana*

## Abstract

We report the molecular characterization of an ethyl methanesulfonate (EMS)-induced mutation that causes albinism and lethality at the seedling stage in *Arabidopsis thaliana*. We identified the mutation using a mapping-by-sequencing approach that uses Fisher’s exact tests to detect changes in allele frequencies among the seedlings of an F_2_ mapping population, which had been pooled according to their phenotypes (wild-type or mutant). After purifying genomic DNA from the plants of both pools, the two samples were sequenced using the Illumina HiSeq 2500 next-generation sequencing platform. The bioinformatic analysis allowed us to identify a point mutation that damages a conserved residue at the acceptor site of an intron of the At2g04030 gene, which encodes the chloroplast-localized AtHsp90.5 protein, a member of the HSP90 family of heat shock proteins. Our RNA-seq analysis demonstrates that the new allele alters the splicing of At2g04030 transcripts in multiple ways, leading to massive deregulation of genes encoding plastid-localized proteins. A search for protein–protein interactions using the yeast two-hybrid method allowed us to identify two members of the GrpE superfamily as potential interactors of AtHsp90.5, as has previously been reported for green algae.

## 1. Introduction

By combining the power of massively parallel sequencing technologies and bulked segregant analysis, mapping-by-sequencing allows researchers to determine the molecular lesions responsible for the phenotypes of induced mutants in a rapid, efficient, and affordable manner [1,2]. Although this approach is most often applied to crosses involving inbred lines of model organisms, it can also be successfully applied to the identification of mutations in crop plants with larger genomes, such as tomato and soybean [3,4]. In this work, we report the genetic and molecular characterization of an albino mutant of *Arabidopsis thaliana*, which we isolated in a forward genetic screen. To map the gene and identify the causal mutation, we followed an approach that takes advantage of the different allele frequencies that are expected in pools of phenotypically wild-type and mutant plants, and applied it to the F_2_ of a cross between the mutant and the wild-type Columbia-0 (Col-0) accession, which occur as a consequence of tight linkage between the mutated locus and closely linked polymorphisms. We found the albino mutant to be affected in a gene encoding a member of the 90 kDa heat shock (Hsp90) protein family.

Hsp90 proteins function as molecular chaperones, either by participating in the de novo folding of other proteins, or by helping to prevent their denaturation. Hsp90 proteins consist of three conserved domains: (1) an N-terminal ATP-binding domain; (2) a central domain responsible for the binding of client proteins; and (3) a dimerization domain at the C-terminal end [5]. The *Arabidopsis thaliana* genome encodes seven Hsp90 proteins: AtHsp90.1, AtHsp90.2, AtHsp90.3, and AtHsp90.4, which are cytosolic; AtHsp90.5 [6], which is located in the chloroplast; AtHsp90.6, which is in the mitochondria; and AtHsp90.7, which is in the endoplasmic reticulum [7]. In this work, we identified a new mutant allele of the At2g04030 gene, which encodes the chloroplastic AtHsp90.5 protein involved in the passage of proteins across chloroplast membranes [8].

## 2. Results and Discussion

### 2.1. Characterization of an EMS-Induced Albino Mutant

**Figure 1:**
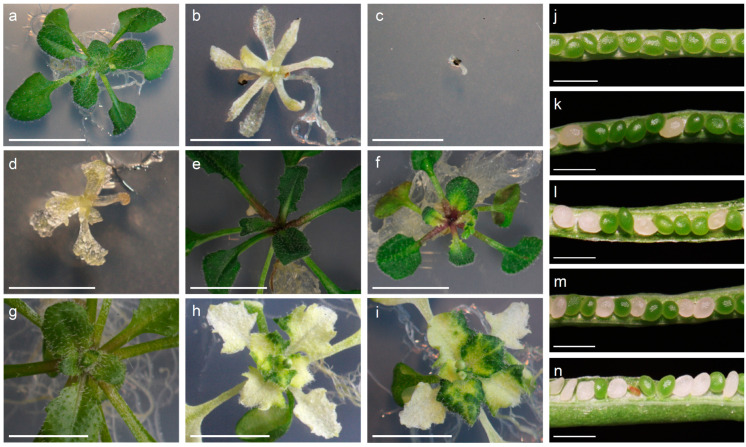
Phenotype of rosettes affected in the function of the At2g04030 (*EMB1956*) gene (**a**–**i**) and segregation of albino embryos in the siliques of plants carrying different mutant alleles of this gene (**j**–**n**). (**a**) Col-0 wild-type. (**b**) Albino plant homozygous for the EMS-induced *emb1956-3* allele grown in MS medium supplemented with sucrose. (**c**) Albino plant homozygous for the *emb1956-3* allele grown in MS medium without sucrose. (**d**) Albino plant from the F_1_ progeny of a complementation cross involving a plant heterozygous for the SALK_012235 allele and the *emb1956-3* allele. Genotyping showed that this plant carries two different mutant alleles of At2g04030. (**e**) Green plant homozygous for the *emb1956-3* allele complemented by the 35S::At2g04030 construct. (**f**) Variegation observed in a plant homozygous for the *emb1956-3* allele due to partial complementation by a 35S::At2g04030 construct. (**g**) Col-0 wild-type. (**h**,**i**) Loss of pigmentation caused by the 35S::At2g04030 construct in a Col-0 background, which has previously been attributed to co-suppression [5]. The pictures were taken at 21 days (**a**), 40 days (**b**,**c**), 25 days (**d**–**f**), and 30 days (**g**–**i**) after sowing. (**j**) Col-0 wild-type. (**k**) *emb1956-3* (EMS-induced allele described in this work). (**l**) SALK_012235. (**m**) SALK_120525. (**n**) CS16104. Scale bars: 5 mm (**a**–**i**) and 1 mm (**j**–**n**).

In an ethyl methanesulfonate (EMS) mutagenesis experiment, we selected an M_3_ family segregating an albino phenotype in embryos and seedlings grown in Murashige-Skoog (MS) medium containing 1% (*m*/*v*) sucrose. In the presence of sucrose, the plants developed poorly beyond the seedling stage and produced a few fully albino leaves (Figure 1b). In the absence of sucrose, by contrast, the seedlings failed to properly expand their cotyledons and did not develop leaves (Figure 1c), as expected if carbon assimilation is defective in the albino mutant. The albino phenotype was inherited as a monogenic recessive trait, as inferred from the 3:1 (wild-type:albino) segregation ratio observed in most of the F_2_ progenies that were studied by counting the albino embryos in developing siliques (Table 1 and Figure 1k). When we scored the phenotype at the seedling stage, by contrast, the albino phenotype appeared in the progeny of self-pollinated heterozygotes at a frequency slightly lower than expected for a monogenic recessive trait (175 wild-type and 32 albino seedlings; χ^2^ > 3.84); this was possibly because of embryonic lethality occurring at low penetrance.

To gain insight into the molecular basis of the albino phenotype, we also obtained F_2_ families by self-pollinating the F_1_ progenies of crosses involving plants heterozygous for the albino mutation and the Col-0 wild-type accession. We then assigned the F_2_ seedlings to two distinct pools based on their phenotypes (wild-type or mutant), and followed a mapping-by-sequencing cloning strategy to identify the mutated gene (see below).

### 2.2. Mapping-By-Sequencing of the Albino Mutant

Sequencing libraries were prepared using DNA from the two pools of F_2_ plants, which, respectively, comprised material from 87 albino and 170 wild-type (green) plants, and were subsequently sequenced using the HiSeq 2500 platform (Illumina) with paired-end 101-bp reads (see Methods). The reads were quality-filtered and aligned to the Col-0 genome using the Bowtie2 read mapper. The resulting alignments (in SAM format) were processed using SAMtools and BCFtools to determine the allele counts at each locus along the genome in the two pools. Aiming to define a set of reliable markers, we focused on all nucleotide positions that were unambiguously biallelic (heterozygous), with individual allele frequencies ranging between 0.15 and 0.85 and sequencing depths between 20 and 120 reads in the pool of phenotypically wild-type plants. We then tabulated the allele counts in the two pools for all subsequent analyses. In a segregating F_2_ mapping population, any marker that is tightly linked to a mutation that results in a recessive trait is expected to have different allele counts between the two pools. In the pool of mutant plants selected from the F_2_ mapping population, the relative frequency of the alleles derived from the mutant progenitor should reach a maximum (i.e., equal to 1) at the site of the mutation, while it should approach one-third in the pool of their wild-type siblings. Reciprocally, the relative frequency of Col-0 alleles (i.e., the allele derived from the wild-type progenitor) is expected to approach zero in the pool of mutants and two-thirds in the pool of wild-type seedlings. At unlinked positions, by contrast, the allele frequencies should be similar and close to 0.5 in the two pools. Mapping-by-sequencing methods typically look for the maximum in the pool of recessive mutants [1].

**Figure 2:**
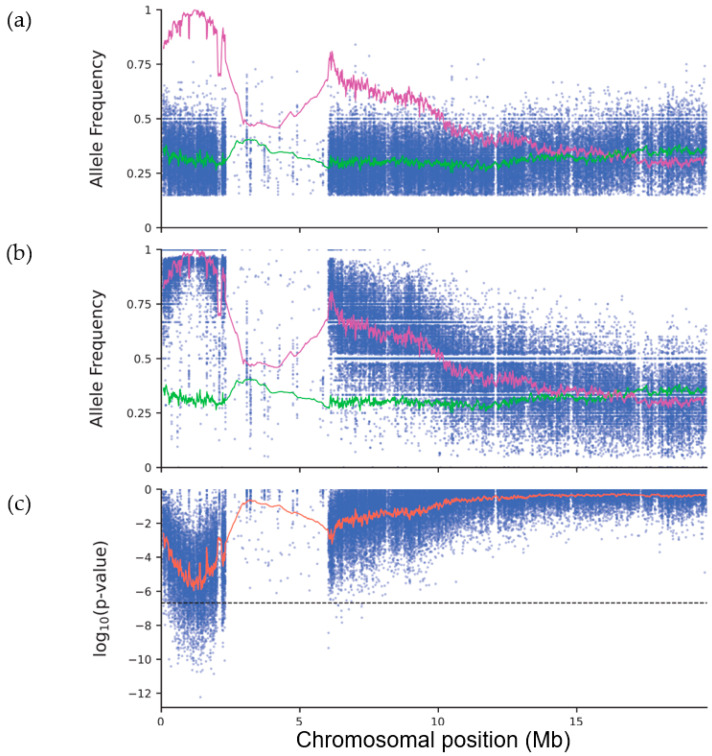
Mapping-by-sequencing of the recessive allele causing the albino seedling phenotype. The plots correspond to markers located on chromosome 2. (**a**) Frequency of alleles inherited from the mutant parent in a pool of F_2_ seedlings exhibiting the wild-type (photosynthetic) phenotype. (**b**) Frequency of alleles inherited from the mutant parent in a pool of F_2_ seedlings exhibiting the mutant (albino) phenotype. (**c**) *p*-values of two-tailed Fisher’s exact tests performed on the same dataset. Each blue dot indicates the allele frequency (**a**,**b**) or the *p*-value (**c**) of a biallelic SNP segregating in the population. The green line corresponds to the moving average of the allele frequencies of 200 adjacent SNPs, as determined in the pool of wild-type plants. The pink line corresponds to the moving average in the pool of mutant individuals. Both moving averages are shown in (**a**,**b**) to facilitate their comparison. The red line corresponds to the weighted moving average of the *p*-values. The dashed line marks the significance threshold, calculated using the Bonferroni correction, considering that n = 237,774 chromosomal locations were tested.

Our dataset comprised allele count data for 237,774 single-nucleotide polymorphic (SNP) markers, which were distributed in the nuclear genome as follows: 44,021 (on chromosome 1), 57,049 (chromosome 2), 51,501 (chromosome 3), 803 (chromosome 4), and 84,400 (chromosome 5). The uneven density and distribution of the polymorphisms along the five chromosomes was expected, because the mutagenized line had a mixed genetic background, which derived from the Landsberg *erecta* (L*er*) accession only in part. For each chromosome, we plotted the frequency of the non-Col-0 allele in the pool of wild-type plants (Figure 2a), which was used as a control, as well as in the pool of albino mutants (Figure 2b). To integrate the values of several adjacent markers and obtain a smoother plot, we also plotted the moving averages of the allele frequencies for all markers placed within a window comprising 200 adjacent markers. The moving average uncovered a peak of maximum allele frequency at position 1,213,007 on chromosome 2 in the pool of mutants (Figure 2b), which was matched by a concomitant reduction in the allele frequency in the pool of wild-type siblings (Figure 2a), indicating that the mutated gene resides on this chromosome. No peaks were observed in the plots for chromosomes 1, 3, 4, and 5 (Figures S1 and S2).

As an alternative approach, we reasoned that any marker linked to the mutated site should also be detected by testing the null hypothesis that the two alleles of any unlinked marker are equally distributed in the two pools. To this end, we used the allele counts from the two pools to calculate the exact probability of obtaining by chance the observed allele counts based on a hypergeometric probability distribution, as well as the *p*-values for two-tailed Fisher’s exact tests (Figure 2c). The lowest *p*-values co-localized to the same chromosomal position as the maximum allele frequency in the pool of mutants; the weighted moving average of the *p*-values reached a minimum value at nucleotide position 1,167,622 on chromosome 2 (Figure 2c). The *p*-values of numerous markers in the vicinity of this position were below the threshold calculated using the Bonferroni correction and the total number of chromosomal locations tested, as expected for markers linked to the albino mutation. No peaks were observed in the corresponding plots of the remaining chromosomes (Figure S3). Fisher’s exact test has previously been applied in the context of QTL-seq [9] and mapping-by-sequencing experiments [10], but its use is not as widespread as the use of methods based on allele frequencies. A remarkable advantage of using probabilities or *p*-values is that they can help to localize a causal mutation, even when the parental origin of each allele is not known, as happens when the sequences of the mutagenized parental strains are not available. Chi-square tests for 2 × 2 contingency tables (given the marginal probabilities for each polymorphic marker) are roughly equivalent to Fisher’s exact tests, and can be used instead as an alternative. Additionally, the statistical tests make no assumption on the expected frequencies of the alleles, which might help to exclude problematic markers that follow a similar distribution in the two pools (e.g., because of incorrect alignments of the reads to the reference, as might be the case for reads derived from repetitive sequences). Based on the position of the markers with extreme *p*-values and allele frequencies, we conservatively defined a wide candidate interval on chromosome 2, between nucleotide positions 1,000,000 and 1,500,000, and searched it for mutations that could explain the observed phenotype.

As an additional way to examine the data, we defined non-overlapping bins containing ten consecutive markers, and pooled their allele counts. These new combined counts were used as a proxy of the haplotype frequencies for each genomic interval, rather than at individual markers. When applied to this new dataset, our analysis pipeline led to identical results, uncovering the effect of higher count numbers on the test’s power to detect linkage between the albino mutation and markers located at distant locations on the same chromosome (Figure S4).

### 2.3. The Albino Mutant Carries a G-To-A Transition Mutation in At2g04030

**Figure 3:**
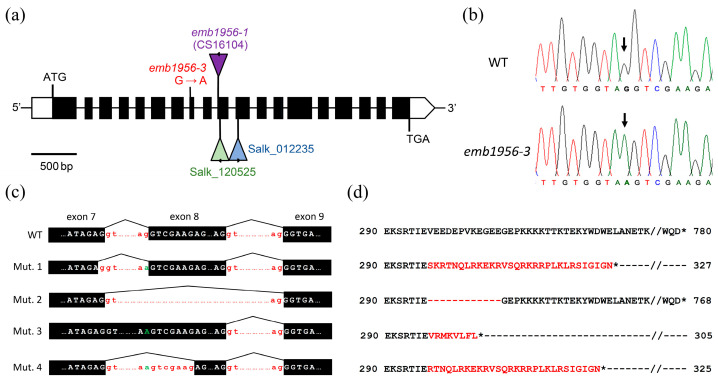
Structure of the At2g04030 gene and effects of the *emb1956-3* mutation described in this work. (**a**) Intron–exon structure of the gene. The black rectangles represent the coding sequences. The 5′ and 3′ untranslated regions (UTRs) are indicated in white. The positions of the insertions and the point mutation are indicated on the scheme. (**b**) G-to-A transition mutation of the *emb1956-3* allele (marked by an arrow), as detected via Sanger sequencing. (**c**) The *emb1956-3* mutation alters the splicing in four different ways, as detected in the RNA-seq experiments. (**d**) The four splice forms are predicted to alter the sequence of their protein products. Asterisks indicate stop codons.

To identify the mutation responsible for the albino phenotype, we made a list with all 2713 genome positions of the candidate interval that, in the pool of mutants, are occupied by a nucleotide other than the one present in the Col-0 genome. This list was screened using several criteria, as previously described [11]. We excluded all the nucleotide substitutions shared with a different mutant line from the same mutagenesis experiment or with the L*er* wild-type, which must correspond to sequence polymorphisms between the genomes of Col-0 and the mutagenized parent. Subsequently, we focused on the G/C-to-A/T transitions present in the candidate interval, since this is the most abundant type of lesion induced by EMS [12]. This filter reduced the number of candidate positions to two G-to-A transition mutations, located at positions 1,100,313 and 1,283,497. The first one was located in an intergenic region, and hence, we considered it less likely to cause the albino phenotype. The second mutation (position 1,283,497 on chromosome 2) was found to damage the last nucleotide of intron 7 in the At2g04030 gene, which corresponds to the highly conserved position −1 of its acceptor (3′) splice site (Figure 3a,b). At2g04030 encodes the plastid-localized AtHsp90.5 protein of the Hsp90 family of heat shock proteins [5,7,8]. Based on the known function and subcellular localization of AtHsp90.5, we pursued this transition mutation as the candidate to explain the albino phenotype. We amplified the genomic region that contains the candidate mutation in 19 albino seedlings, using the At2g04030-F and At2g04030-R primers (Table S1). The PCR products were purified and Sanger-sequenced, and all the albino seedlings were found to be homozygous for the candidate mutation (Figure 3b). These data place the At2g04030 gene and the mutation responsible for the albino phenotype in the same locus on chromosome 2 (at 0% recombination, since no recombination events were detected in 38 chromosomes).

### 2.4. Complementation Tests Show That the Albino Mutant Is Affected in At2g04030

Based on the phenotypic effects of its mutant alleles, the At2g04030 gene was previously named *EMBRYO DEFECTIVE 1956* (*EMB1956*) [13] and *CHLORATE RESISTANT 88* (*CR88*) [7,8]. Feng and colleagues [7] reported alleles that cause an albino phenotype like that of our mutant. Following the nomenclature used in the TAIR database, we named our EMS-induced allele *emb1956-3*. To confirm that we correctly identified the gene, we selected three independently isolated lines that carry T-DNA insertions in the At2g04030 gene: CS16104 (*emb1956-1*), SALK_012235, and SALK_120525. The developing siliques of plants heterozygous for these three insertion lines contained albino embryos in a 3:1 (green:albino) ratio (Table 1; Figure 1j,l–n). This result is in line with the observation that the SALK mutants have previously been reported to be embryo-lethal, as the albino embryos typically develop into collapsed mature seeds that are unable to germinate [7]. We amplified the sequences flanking the left border (LB) of each T-DNA insertion using primers from Table S1, and determined their exact insertion sites using Sanger sequencing. We performed reciprocal complementation crosses between plants carrying the EMS-induced *emb1956-3* mutation and each of the T-DNA lines. Collapsed seeds were present in all the F_1_ progenies (Table 2), demonstrating that *emb1956-3* is a new loss-of-function allele of the At2g04030 gene. In the presence of sucrose, some F_1_ seeds developed into albino plants (Figure 1d), which were genotyped to confirm the presence of mutant alleles from both parents.

### 2.5. Complementation Using a Transgene

In addition to the above-mentioned crosses, we also made a construct to overexpress the AtHsp90.5 protein under the control of a constitutive 2x35S promoter (pMDC32-At2g04030; see Methods). The construct was transformed into the Col-0 wild-type accession, and multiple independent transformants were selected in MS medium supplemented with hygromycin (Figure 1g–i). In some transformants, the transgene caused variegated pigmentation in the rosette leaves (Figure 1h,i), a phenotype that has been previously attributed to co-suppression (post-transcriptional silencing) of the endogenous gene [5]. Other transformants (in which the expression of the transgene was confirmed by RT-PCR) exhibited a wild-type phenotype and were crossed with plants heterozygous for the EMS-induced allele to rescue the defects caused by the *emb1956-3* allele. In the F_2_ progeny of these crosses, we isolated plants that were homozygous for the albino mutation and carried at least one copy of the transgene (Figure 1e,f). These plants were green, showing that the transgene can complement the effects of the EMS-induced allele (Figure 1e), but some of them eventually turned albino, suggesting that the transgene is silenced at later developmental stages (Figure 1f).

### 2.6. The Albino Mutation Causes Massive Downregulation of Chloroplast-Related Functions

To further understand the effects of the *emb1956-3* mutation on above-ground tissues, we performed an RNA sequencing (RNA-seq) experiment to compare the gene expression levels in albino and wild-type plants. To compensate for the effects of the genetic background and the slower growth of the mutant plants, above-ground tissue was collected at roughly equivalent developmental stages from wild-type and mutant siblings of the same segregating family. By setting the false discovery rate (FDR) threshold at 5%, we detected altered expression levels for 9005 genes in the albino mutant, including 4504 downregulated (Table S2) and 4501 upregulated genes (Table S3). To assign Gene Ontology (GO) terms to each gene, and to identify GO terms overrepresented in the sets of downregulated and upregulated genes, we used the PANTHER Classification System [14]. The PANTHER Classification System assigned 9983 GO terms to 19000 genes (out of the 20144 genes expressed in our samples that were selected as the custom background set), including 1024 terms from the ‘cellular component’ (CC) subontology, 5789 terms from the ‘biological process’ (BP) subontology, and 3170 terms from the ‘molecular function’ (MF) subontology. The GO terms were tested separately for overrepresentation in the sets of upregulated and downregulated genes (Tables S4 and S5).

In the set of downregulated genes, most of the significantly enriched CC terms were related to the plastids/chloroplasts and their constituent parts, as one would expect for a mutant with severely impaired chloroplast function. In fact, the availability of other hypomorphic alleles has previously allowed for the characterization of the plastid ultrastructure of albino plants using transmission electron microscopy (TEM), which uncovered severe defects in their thylakoidal membranes [7]. In line with these defects, the most highly enriched CC terms included terms such as ‘photosystem I reaction center’ (GO:0009538), ‘chloroplastic endopeptidase Clp complex’ (GO:0009840), and ‘plastid thylakoid lumen’ (GO:0031978), which had fold enrichment factors above 4. Most terms from the BP subontology were also related to processes specific to the plastids, such as ‘photosynthesis’ (GO:0015979) and ‘plastid organization’ (GO:0009657), followed by other terms related to biosynthetic pathways that occur in the chloroplasts. A reciprocal trend was observed in the set of upregulated genes as regards chloroplast-related terms, many of which appeared significantly underrepresented, indicating that the loss of AtHsp90.5 activity leads to the drastic downregulation of chloroplast-related functions. The data in Table S2 show massive downregulation of genes encoding subunits of ribulose-1,5-bisphosphate carboxylase-oxygenase and many other components of the photosynthetic complexes, which are normally expressed at remarkably high levels.

In the set of upregulated genes, by contrast, we found many overrepresented GO terms related to translation and the biogenesis and structure of cytosolic ribosomes. A similar result was previously described for the *orbiculata1* mutants [15], in which the concerted upregulation of genes encoding ribosomal proteins was observed as a consequence of defective glutamine biosynthesis in the plastids. Because the genes encoding subunits of photosynthetic complexes and ribosomes together make up the most abundant mRNA populations in the cell, we speculate that a drastic reduction in the transcript levels of photosynthesis-related genes might be detected as an apparent increase in the relative transcript levels of other housekeeping genes, such as those encoding structural components of the cytosolic ribosome. Among the genes with the highest fold changes, we also found two *HYPOXIA-RESPONSIVE UNKNOWN PROTEIN* (*HUP*) genes, *HUP7* and *HUP44* [16,17], in line with the overrepresentation of GO terms related to the response to O_2_ deprivation (e.g., GO:0071456 ‘cellular response to hypoxia’) in this set. In a recent study, Islam and collaborators [18] investigated the changes in gene expression upon induced silencing of the *HSP90C* gene using transgenic tobacco plants. Despite the differences in the experimental setup, we found that 34.2% of the genes downregulated in Arabidopsis (i.e., 1540 genes out of the 4504 downregulated genes) were also downregulated in the tobacco system, but only 7.7% of the genes upregulated in Arabidopsis (i.e., 552 out of 4501 upregulated genes) were also upregulated in tobacco.

### 2.7. The Transition Mutation Causes Improper Splicing of At2g04030 Transcripts

Because the transition mutation alters a highly conserved residue in the acceptor splice site of intron 7, we amplified the cDNA of the At2g04030 gene from *emb1956-3* and wild-type plants using the primers SALK_120525-LP and At2g04030-R (Table S1), in an attempt to detect improperly spliced transcripts. Sequencing of the amplification products uncovered a frameshift starting at the last codon of exon 7 (Figure 3c,d, Mut. 1), due to incorrect use of the 5′ (donor) splice site of intron 7 (i.e., the intron was cleaved one nucleotide upstream of the normal site, within the sequence of exon 7).

We took advantage of our RNA-Seq data to detect and determine the abundance of aberrant splice forms (Figure 3c) in the albino mutant. The form identified in the RT-PCR experiments was the most abundant one, supported by 87% of the reads mapped to the 3′ end of exon 7 (456 reads out of 524 total reads, Figure 3c, Mut. 1), and was predicted to encode a truncated protein of 327 amino acids (Figure 3d). We also detected other forms, resulting from events such as: (1) the skipping of exon 8 (Figure 3c, Mut. 2), which was supported by 7.4% of the reads (39 reads) and is predicted to cause the in-frame deletion of 12 amino acids (Figure 3d); (2) the retention of intron 7 (Figure 3c, Mut. 3), which was supported by 5% of the reads (26 reads) and creates a frameshift that leads to premature truncation of the protein (Figure 3d); and (3) the rare use of a cryptic acceptor site within the sequence of exon 8 (Figure 3c, Mut. 4), which was supported by the remaining 0.6% of the reads (3 reads) and is predicted to create a frameshift (Figure 3d). In conclusion, all the observed splice forms are expected to alter the sequence and/or truncate the protein, linking the observed phenotype to a change in the coding potential of the mutant transcripts.

### 2.8. Identification of New Protein–Protein Interactions Involving the AtHsp90.5 Protein

To further our understanding of the roles played by *AtHsp90.5* in the chloroplasts of *A. thaliana*, we prepared a bait construct containing its full-length coding sequence and performed a yeast two-hybrid (Y2H) screen against a cDNA library derived from multiple tissues (Figure S5). In this screen, we isolated a prey clone corresponding to the At1g36390 gene, also known as *CHLOROPLAST GRPE 2* (*CGE2*), which encodes a member of the GrpE superfamily. Our sequence analysis indicates that the At1g36390 coding sequence is fused to the correct frame of the activation domain (AD) of Gal4 in this prey clone (Figure S5). Although, to our knowledge, an interaction between AtHsp90.5 and At1g36390 has not been reported yet for any flowering plant, the homologs of these two proteins have previously been reported to interact in *Chlamydomonas reinhardtii* [19]. So far, our attempts to reproduce this interaction using prey and bait constructs containing the full-length coding sequence of At1g36390 in reciprocal Y2H assays have failed. We also tested the interaction between AtHsp90.5 and constructs containing the full-length coding sequence of At5g17710 (also known as *EMBRYO DEFECTIVE 1241* (*EMB1241*) and *CHLOROPLAST GRPE 1* (*CGE1*)), which is the only paralog of At1g36390 in the *A. thaliana* genome [20]. The proteins encoded by At1g36390 and At5g17710 are thought to participate in the correct oligomerization of light-harvesting complex II (LHCII) and the response to heat stress. Our Y2H assays allowed us to detect an interaction between AtHsp90.5 and At5g17710 only when using At5g17710 as bait and AtHsp90.5 as prey, but not in the reciprocal configuration (Figure S5).

## 3. Materials and Methods

### 3.1. Plant Material and Growth of A. thaliana Plants

The albino mutant was identified in an M_3_ family after the EMS mutagenesis of *A. thaliana* seeds, following a previously described protocol [21]. For the study of the mutant phenotype and the segregation ratios, the seeds were surface-sterilized and stratified at 4 °C for 24 h to synchronize their germination, and then, sown on Petri dishes containing half-strength Murashige and Skoog (MS) solid medium [22] containing 1% (*m*/*v*) sucrose. The plates were incubated at 20 ± 0.1 °C in an HLR-352-PE (Panasonic) incubator with continuous illumination supplied by Panasonic 40 FL40SS-ENW-37 fluorescent tubes. At the onset of bolting (i.e., about 21 days after sowing), the plants were transferred to soil and were allowed to complete their life cycle in a growth chamber. T-DNA lines were obtained from the Nottingham Arabidopsis Stock Centre collection.

### 3.2. Genome Sequencing and Bioinformatic Analysis

Genome sequencing was conducted by STAB VIDA (Caparica, Portugal) using an Illumina HiSeq 2500 massively parallel sequencer. Fragments were sequenced using paired-end 101 bp reads. Read quality and the presence of adapter sequences were assessed using FastQC (http://www.bioinformatics.babraham.ac.uk/projects/fastqc/, accessed on 19 February 2023). The reads were then processed using Trimmomatic (version 0.39) [23], with the following options: ILLUMINACLIP:TruSeq3-PE.fa:2:30:10:2:keepBothReads, LEADING:3, TRAILING:3, and MINLEN:36. The TAIR10 version of the *A. thaliana* genome was used as the reference genome [24]. The read pairs were aligned to the reference genome using Bowtie2 (version 2.4.5) [25,26], with the following options: --X 1000, --no-discordant, --no-mixed, and --no-unal. The resulting files were converted from SAM (sequence alignment/map) format to BAM (binary alignment map) format using the view command of SAMtools version 1.16.1 [27], and the resulting BAM files were sorted according to the location of the reads in the reference genome using the sort command. The base calling and identification of variants were performed using the mpileup (with the options -f and --annotate FORMAT/AD) and call (with the options -m and -V indels) commands of BCFtools version 1.16 [28], and stored as VCF (variant call format) files [29]. The allele counts were subsequently used to calculate allele frequencies and *p*-values along all chromosomes.

### 3.3. Peak Detection

In addition to the allele frequencies, we also calculated the *p*-values of exact Fisher’s tests using the observed allele counts, as implemented in the Python SciPy library [30]. The *p*-values for individual markers were plotted against their chromosomal locations. To integrate the data of a window containing *k* adjacent markers, we also calculated the weighted geometric mean of their *p*-values, using their sequencing depths as weights (i.e., the number n of reads covering each individual marker).

### 3.4. Constructs for Plant Transformation and Yeast Two-Hybrid Assays

We amplified the coding region of the At2g04030 gene with the primers At2g04030-cds-F and At2g04030-cds-R, using cDNA from Columbia-0 (Col-0) basal rosettes as the template. The PCR product was cloned in the pGEM-T Easy 221 vector using Gateway cloning technology, and then, transferred to the pMDC32 Gateway destination vector. The pMDC32-At2g04030 construct carries two tandem copies of the CaMV 35S promoter, which drives the constitutive expression of the At2g04030 gene. This construct was transformed into *Agrobacterium tumefaciens*, which allowed us to transform wild-type Col-0 plants and plants heterozygous for the albino mutation using the floral dip method [31].

In addition, we used the same entry clone (in pGEM-T Easy 221) to transfer the coding region of the At2g04030 gene to the pDEST-GBKT7 Gateway destination vector. The resulting pDEST-GBKT7-At2g04030 construct contained an in-frame fusion of the GAL4 DNA-binding domain and the At2g04030 protein.

Using the At1g36390-cds-F/At1g36390-cds-R and At5g17710-cds-F/At5g17710-cds-R primers, we amplified the full-length coding regions of the At1g36390 and At5g17710 genes using cDNA from Col-0 rosettes as the template. The PCR products were cloned in pGEM-T Easy 221, sequenced, and subsequently transferred to appropriate destination vectors for yeast two-hybrid assays.

### 3.5. RNA Sequencing

RNA was purified using the MagMAX™ Plant RNA Isolation Kit (Thermo Fisher Scientific, Rockford, IL, USA) for three biological replicates of each experimental condition. Paired-end libraries were prepared using the TruSeq Stranded mRNA LT Sample Prep Kit and sequenced using the NovaSeq platform (Illumina) by Macrogen (Seoul, Republic of Korea). Reads (151 bp long) were aligned to an index based on the TAIR10 reference genome sequence and the Araport11 annotation [32] using HISAT2 (version 2.2.1) [33], with the options: --no-discordant, --no-mixed, --dta-cufflinks, and --rna-strandness RF. The genes included in Araport11 were tested for differential expression using Cuffdiff (version 2.2.1) [34], with the library type set to fr-firststrand.

For the statistical evaluation of term overrepresentation, we used the PANTHER Overrepresentation Test tool to perform Fisher’s exact tests with FDR correction, using the annotation of the GO Ontology database (DOI:10.5281/zenodo.4495804, released on 1 February 2021). Tests were performed separately for the three different subontologies, using the set of genes expressed in our samples (those marked “OK” by Cuffdiff) as the custom reference.

### 3.6. Yeast Two-Hybrid Screen

Yeast two-hybrid (Y2H) screening was conducted using the Matchmaker^TM^ GAL4 Two-Hybrid System (Clontech Laboratories Inc., Mountain View, CA, USA), with a normalized cDNA library prepared in the pGADT7-RecAB vector using mRNA isolated from eleven tissues of *Arabidopsis thaliana* (Mate & Plate^TM^ Library—Universal Arabidopsis (Normalized), Takara Bio USA, Inc., Mountain View, CA, USA). A bait construct (pDEST-GBKT7-At2g04030) was transformed into the yeast strain Y2HGold and mated to the library (in yeast strain Y187). We also prepared bait and prey constructs containing the full-length coding sequences of *AtHsp90*.5 (At2g04030) and the two GrpE paralogs (At1g36390 and At5g17710) in pDest-GBKT7 and pDest-GADT7. For the reciprocal pairwise Y2H assays, constructs in pDest-GBKT7 and pDest-GADT7 were transformed into the strains Y2HGold and Y187, respectively.

## 4. Conclusions

The main advantage of mapping-by-sequencing methods is that they allow for rapid determination of the chromosomal location for a gene of interest, while simultaneously leading to identification of the mutation that causes the mutant phenotype [2]. In these methods, a causal mutation is typically identified because it colocalizes with the peak of maximum allele frequency in a pool of plants displaying the recessive phenotype. Although similar principles have been applied to a related approach called QTL-seq, a variety of other statistics have been developed to assist in the detection and mapping of quantitative trait loci (QTLs) [35]. In this manuscript, we show that Fisher’s exact tests are an alternative, complementary method to map the mutation underlying a phenotype of interest in a mapping-by-sequencing experiment. After defining an interval containing two candidate mutations, we used complementation crosses and transgenic plants to unequivocally demonstrate that we had correctly identified the EMS-induced mutation responsible for the albino phenotype observed in developing embryos and seedlings. Additionally, our RNA-seq results helped us to explain how the identified nucleotide substitution alters the splicing of mutant transcripts in multiple ways, disrupting the function of the encoded protein in all cases, and to gain insight on the massive downregulation of nuclear genes encoding chloroplast-localized proteins that occurs when the function of the plastid-localized AtHsp90.5 protein is compromised.

## Figures and Tables

**Table 1:** Segregation of albino embryos in F_2_ families segregating mutant alleles of the At2g04030 gene.

Allele	Family	Wild-Type	Albino	χ^2^	*p*-Value ^1^
*emb1956-3* (this work)	1	49	19	0.314	0.575
	2	61	30	3.081	0.079
	3	67	16	1.450	0.229
	4	46	32	10.684	0.001 *
SALK_120525	1	54	15	0.391	0.532
	2	96	35	0.206	0.650
	3	93	31	0.000	1.000
	4	125	51	1.485	0.223
CS16104	1	119	41	0.033	0.855
	2	90	39	1.884	0.170
	3	119	44	0.346	0.557
	4	126	52	1.685	0.194
SALK_012235	1	86	32	0.282	0.595
	2	117	35	0.316	0.574
	3	104	37	0.116	0.734
	4	130	53	1.532	0.216

^1^ One-tailed *p*-values from χ^2^ goodness-of-fit tests of the observed data to the expected 3:1 segregation ratio. * Significant at 5% level (1 degree of freedom).

**Table 2:** Segregation of collapsed seeds in F_1_ progenies of complementation crosses.

Cross	Wild-Type	Albino	χ^2^	*p*-Value *
CS16104 × *EMB1956*/*emb1956-3*	45	23	2.824	0.093
SALK_012235 × *EMB1956*/*emb1956-3*	46	16	0.022	0.883
SALK_120525 × *EMB1956*/*emb1956-3*	28	9	0.009	0.924
*EMB1956*/*emb1956-3* × CS16104	22	10	0.667	0.414
*EMB1956*/*emb1956-3* × SALK_012235	49	9	2.782	0.095

* One-tailed *p*-values from χ^2^ goodness-of-fit tests of the observed data to the expected 3:1 segregation ratio. Due to the embryonic lethality, all the plants selected for the crosses were heterozygous for mutant alleles.2.5. Complementation Using a Transgene

## Data Availability

The raw reads for the mapping-by-sequencing and RNA-seq experiments have been submitted to the BioProject database with accession numbers PRJNA934907 and PRJNA935322, respectively.

## Supplementary Materials

The following supporting information can be downloaded at: https://www.mdpi.com/article/10.3390/ijms24044196/s1.

## References

[1] Schneeberger K., Ossowski S., Lanz C., Juul T., Petersen A.H., Nielsen K.L., Jørgensen J.-E., Weigel D., Andersen S.U. (2009). SHOREmap: Simultaneous Mapping and Mutation Identification by Deep Sequencing. Nat. Methods.

[2] Candela H., Casanova-Sáez R., Micol J.L. (2015). Getting Started in Mapping-by-Sequencing. J. Integr. Plant Biol..

[3] Li Z., Guo Y., Ou L., Hong H., Wang J., Liu Z., Guo B., Zhang L., Qiu L. (2018). Identification of the Dwarf Gene GmDW1 in Soybean (*Glycine max* L.) by Combining Mapping-by-Sequencing and Linkage Analysis. Theor. Appl. Genet..

[4] Takei H., Shinozaki Y., Yano R., Kashojiya S., Hernould M., Chevalier C., Ezura H., Ariizumi T. (2019). Loss-of-Function of a Tomato Receptor-Like Kinase Impairs Male Fertility and Induces Parthenocarpic Fruit Set. Front. Plant Sci..

[5] Oh S.E., Yeung C., Babaei-Rad R., Zhao R. (2014). Cosuppression of the Chloroplast Localized Molecular Chaperone HSP90.5 Impairs Plant Development and Chloroplast Biogenesis in Arabidopsis. BMC Res. Notes.

[6] Cao D., Froehlich J.E., Zhang H., Cheng C.-L. (2003). The Chlorate-Resistant and Photomorphogenesis-Defective Mutant Cr88 Encodes a Chloroplast-Targeted HSP90. Plant J..

[7] Feng J., Fan P., Jiang P., Lv S., Chen X., Li Y. (2014). Chloroplast-Targeted Hsp90 Plays Essential Roles in Plastid Development and Embryogenesis in Arabidopsis Possibly Linking with VIPP1. Physiol. Plant..

[8] Inoue H., Li M., Schnell D.J. (2013). An Essential Role for Chloroplast Heat Shock Protein 90 (Hsp90C) in Protein Import into Chloroplasts. Proc. Natl. Acad. Sci. USA.

[9] Yang L., Wang J., Han Z., Lei L., Liu H.L., Zheng H., Xin W., Zou D. (2021). Combining QTL-Seq and Linkage Mapping to Fine Map a Candidate Gene in QCTS6 for Cold Tolerance at the Seedling Stage in Rice. BMC Plant Biol..

[10] Fekih R., Takagi H., Tamiru M., Abe A., Natsume S., Yaegashi H., Sharma S., Sharma S., Kanzaki H., Matsumura H. (2013). MutMap+: Genetic Mapping and Mutant Identification without Crossing in Rice. PLoS ONE.

[11] Mateo-Bonmatí E., Casanova-Sáez R., Candela H., Micol J.L. (2014). Rapid Identification of Angulata Leaf Mutations Using Next-Generation Sequencing. Planta.

[12] Greene E.A., Codomo C.A., Taylor N.E., Henikoff J.G., Till B.J., Reynolds S.H., Enns L.C., Burtner C., Johnson J.E., Odden A.R. (2003). Spectrum of Chemically Induced Mutations From a Large-Scale Reverse-Genetic Screen in Arabidopsis. Genetics.

[13] Tzafrir I., Pena-Muralla R., Dickerman A., Berg M., Rogers R., Hutchens S., Sweeney T.C., McElver J., Aux G., Patton D. (2004). Identification of Genes Required for Embryo Development in Arabidopsis. Plant Physiol..

[14] Mi H., Muruganujan A., Casagrande J.T., Thomas P.D. (2013). Large-Scale Gene Function Analysis with the PANTHER Classification System. Nat. Protoc..

[15] Muñoz-Nortes T., Pérez-Pérez J.M., Sarmiento-Mañús R., Candela H., Micol J.L. (2017). Deficient Glutamate Biosynthesis Triggers a Concerted Upregulation of Ribosomal Protein Genes in Arabidopsis. Sci. Rep..

[16] Mustroph A., Lee S.C., Oosumi T., Zanetti M.E., Yang H., Ma K., Yaghoubi-Masihi A., Fukao T., Bailey-Serres J. (2010). Cross-Kingdom Comparison of Transcriptomic Adjustments to Low-Oxygen Stress Highlights Conserved and Plant-Specific Responses. Plant Physiol..

[17] Branco-Price C., Kawaguchi R., Ferreira R.B., Bailey-Serres J. (2005). Genome-Wide Analysis of Transcript Abundance and Translation in Arabidopsis Seedlings Subjected to Oxygen Deprivation. Ann. Bot..

[18] Islam S., Bhor S.A., Tanaka K., Sakamoto H., Yaeno T., Kaya H., Kobayashi K. (2020). Impaired Expression of Chloroplast HSP90C Chaperone Activates Plant Defense Responses with a Possible Link to a Disease-Symptom-Like Phenotype. Int. J. Mol. Sci..

[19] Willmund F., Dorn K.V., Schulz-Raffelt M., Schroda M. (2008). The Chloroplast DnaJ Homolog CDJ1 of Chlamydomonas Reinhardtii Is Part of a Multichaperone Complex Containing HSP70B, CGE1, and HSP90C. Plant Physiol..

[20] de Luna-Valdez L.A., Villaseñor-Salmerón C.I., Cordoba E., Vera-Estrella R., León-Mejía P., Guevara-García A.A. (2019). Functional Analysis of the Chloroplast GrpE (CGE) Proteins from *Arabidopsis thaliana*. Plant Physiol. Biochem..

[21] Kim Y., Schumaker K.S., Zhu J.-K., Salinas J., Sanchez-Serrano J.J. (2006). EMS Mutagenesis of Arabidopsis. Arabidopsis Protocols.

[22] Murashige T., Skoog F. (1962). A Revised Medium for Rapid Growth and Bio Assays with Tobacco Tissue Cultures. Physiol. Plant..

[23] Bolger A.M., Lohse M., Usadel B. (2014). Trimmomatic: A Flexible Trimmer for Illumina Sequence Data. Bioinformatics.

[24] Lamesch P., Berardini T.Z., Li D., Swarbreck D., Wilks C., Sasidharan R., Muller R., Dreher K., Alexander D.L., Garcia-Hernandez M. (2012). The Arabidopsis Information Resource (TAIR): Improved Gene Annotation and New Tools. Nucleic Acids Res..

[25] Langmead B., Trapnell C., Pop M., Salzberg S.L. (2009). Ultrafast and Memory-Efficient Alignment of Short DNA Sequences to the Human Genome. Genome Biol..

[26] Langmead B., Salzberg S.L. (2012). Fast Gapped-Read Alignment with Bowtie 2. Nat. Methods.

[27] Li H., Handsaker B., Wysoker A., Fennell T., Ruan J., Homer N., Marth G., Abecasis G., Durbin R. (2009). 1000 Genome Project Data Processing Subgroup The Sequence Alignment/Map Format and SAMtools. Bioinformatics.

[28] Danecek P., Bonfield J.K., Liddle J., Marshall J., Ohan V., Pollard M.O., Whitwham A., Keane T., McCarthy S.A., Davies R.M. (2021). Twelve Years of SAMtools and BCFtools. GigaScience.

[29] Danecek P., Auton A., Abecasis G., Albers C.A., Banks E., DePristo M.A., Handsaker R.E., Lunter G., Marth G.T., Sherry S.T. (2011). The Variant Call Format and VCFtools. Bioinformatics.

[30] Virtanen P., Gommers R., Oliphant T.E., Haberland M., Reddy T., Cournapeau D., Burovski E., Peterson P., Weckesser W., Bright J. (2020). SciPy 1.0: Fundamental Algorithms for Scientific Computing in Python. Nat. Methods.

[31] Clough S.J., Bent A.F. (1998). Floral Dip: A Simplified Method for Agrobacterium -Mediated Transformation of *Arabidopsis thaliana*. Plant J..

[32] Cheng C.-Y., Krishnakumar V., Chan A.P., Thibaud-Nissen F., Schobel S., Town C.D. (2017). Araport11: A Complete Reannotation of the *Arabidopsis thaliana* Reference Genome. Plant J..

[33] Kim D., Paggi J.M., Park C., Bennett C., Salzberg S.L. (2019). Graph-Based Genome Alignment and Genotyping with HISAT2 and HISAT-Genotype. Nat. Biotechnol..

[34] Trapnell C., Hendrickson D.G., Sauvageau M., Goff L., Rinn J.L., Pachter L. (2013). Differential Analysis of Gene Regulation at Transcript Resolution with RNA-Seq. Nat. Biotechnol..

[35] de la Fuente Cantó C., Vigouroux Y. (2022). Evaluation of Nine Statistics to Identify QTLs in Bulk Segregant Analysis Using next Generation Sequencing Approaches. BMC Genomics.

