# Supplementary material for: Cloning of an Albino Mutation of Arabidopsis thaliana Using Mapping-by-Sequencing
Source: Int J Mol Sci. 2023 Feb 20;24(4):4196. doi: 10.3390/ijms24044196 (PMC9964061; doi:10.3390/ijms24044196)
Supplement: Supplementary file 1 [file ijms-24-04196-s001.zip › ijms-2115695-supplementaryREVISED/Supplemental Materials v2.pdf]

Table S1. Oligonucleotides used in this work.

Primer	Nucleotide sequence (5′→3′)
At2g04030-F	CGTCGTGGAACACAAATCAC
At2g04030-R	AGGCTTACCCATAGCGGTTT
At2g04030-cds-F	ggggacaagtttgtacaaaaaagcaggctTAATGGCTCCTGCTTTGAGTA
At2g04030-cds-R	ggggaccactttgtacaagaaagctgggtCAATCTTGCCAAGGATCACTC
At5g17710-cds-F	ggggacaagtttgtacaaaaaagcaggctCCATGGCCGGTCTACTCAAAA
At5g17710-cds R1	ggggaccactttgtacaagaaagctgggtCAAGATGAAGATGATTCTTC
At1g36390-cds-F	ggggacaagtttgtacaaaaaagcaggctCAATGGCGATTTCTTTCGCAA
At1g36390-cds R1	ggggaccactttgtacaagaaagctgggtCAAGCAGAAGGTGTTATTTC
SP6	ATTTAGGTGACACTATAG
T7 Fwd pGEM	GTAATACGACTCACTATAGGGC
M13-F	CGCCAGGGTTTTCCCAGTCACGAC
M13-R	TCACACAGGAAACAGCTATGAC
NOS-R	AGTAACATAGATGACACCGCGC
CS16104-F	TCTCAGTTCGTTGGGTTTCC
CS16104-R	CACGCTTCACGTAGAGACGA
Salk_012235 RP	ATTGGGATTGGGAACTAGCC
Salk_012235 LP	TCATCCAAGCTTGTCAATTC
Salk_120525 RP	TCATCAGAGTCCACAACTCCC
Salk_120525 LP	GGTTGTTGTGTCCACCAAAAG
SALK_LBb1.3	ATTTTGCCGATTTCGGAAC
SAIL_LB3	TAGCATCTGAATTTCATAACCAATCTCGATACAC
Oligo (dT)18	TTTTTTTTTTTTTTTTTT
T7 universal	TAATACGACTCACTATAGGG
3AD primer	AGATGGTGCACGATGCACAG

* The sequences of the attB1 and attB2 sites, used by the Gateway cloning technology, are indicated using lowercase characters.

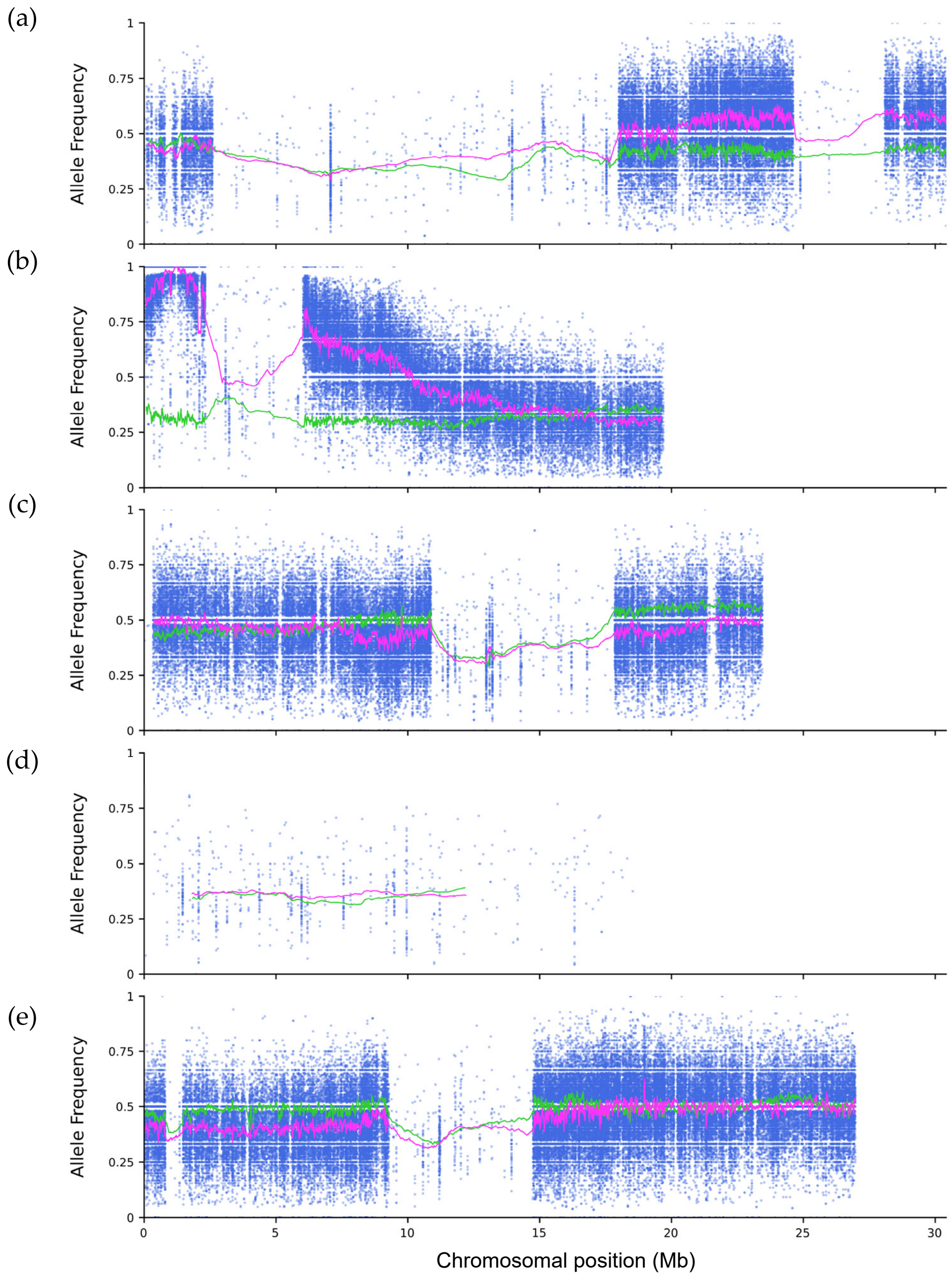

Figure S1. Frequency of alleles inherited from the mutant parent in a pool of F_2 seedlings exhibiting the mutant (albino) phenotype. (a) Chromosome 1. (b) Chromosome 2. (c) Chromosome 3. (d) Chromosome 4. (e) Chromosome 5. Each blue dot indicates the allele frequency of a biallelic SNP segregating in the population, as determined for the pool of mutants. The pink line indicates the moving average of the allele frequencies of 200 adjacent SNPs. The green line corresponds to the moving average of the allele frequencies in a pool of F_2 plants displaying the wild-type phenotype and is shown for comparison.

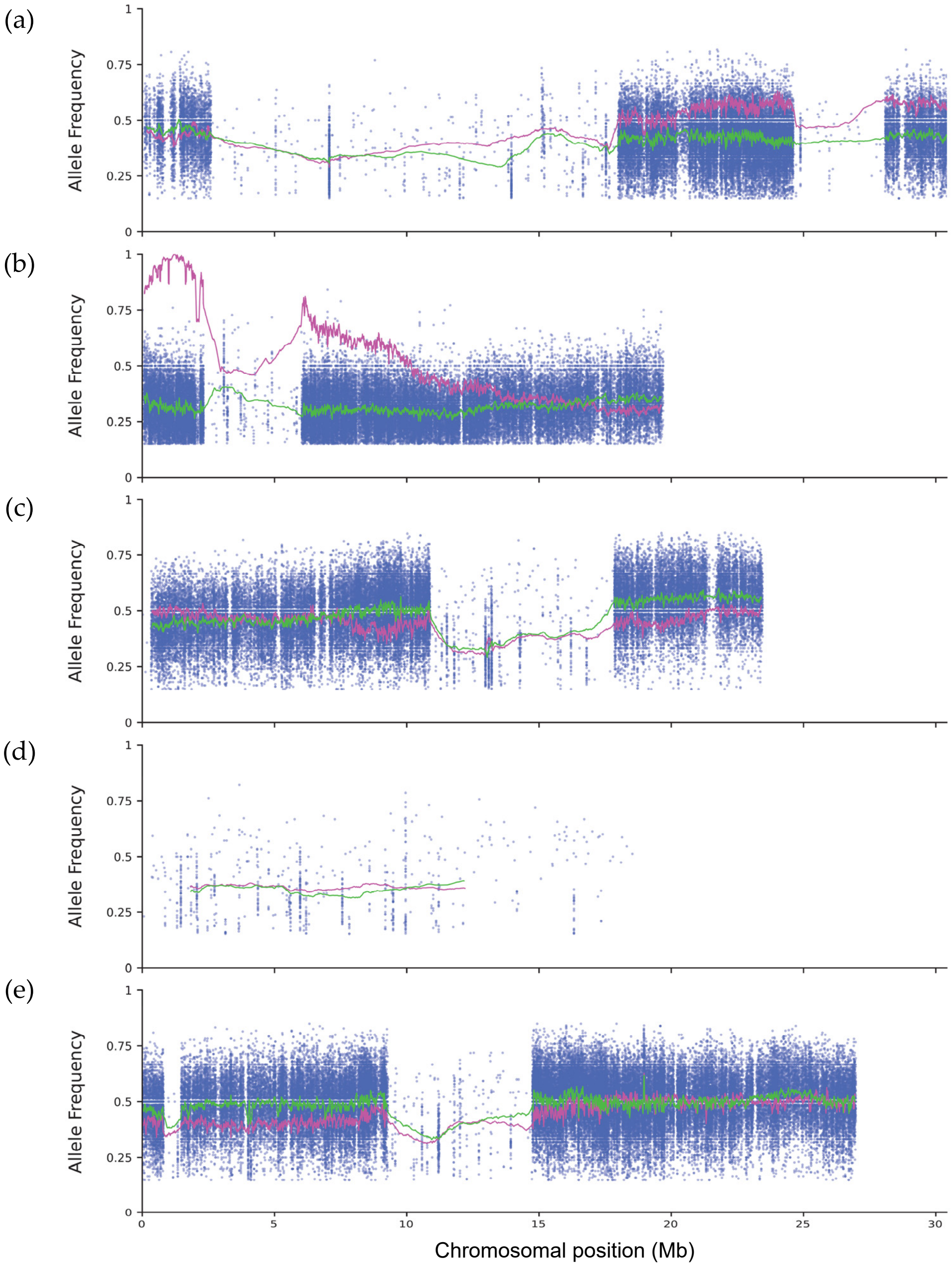

Figure S2. Frequency of alleles inherited from the mutant parent in a pool of F_2 seedlings exhibiting the wild-type (photosynthetic) phenotype. (a) Chromosome 1. (b) Chromosome 2. (c) Chromosome 3. (d) Chromosome 4. (e) Chromosome 5. Each blue dot indicates the allele frequency of a biallelic SNP segregating in the population, as determined for the pool of wild-type siblings. The green line indicates the moving average of the allele frequencies at 200 adjacent SNPs. The pink line corresponds to the moving average of the allele frequencies in a pool of F_2 plants displaying the mutant phenotype and is shown for comparison.

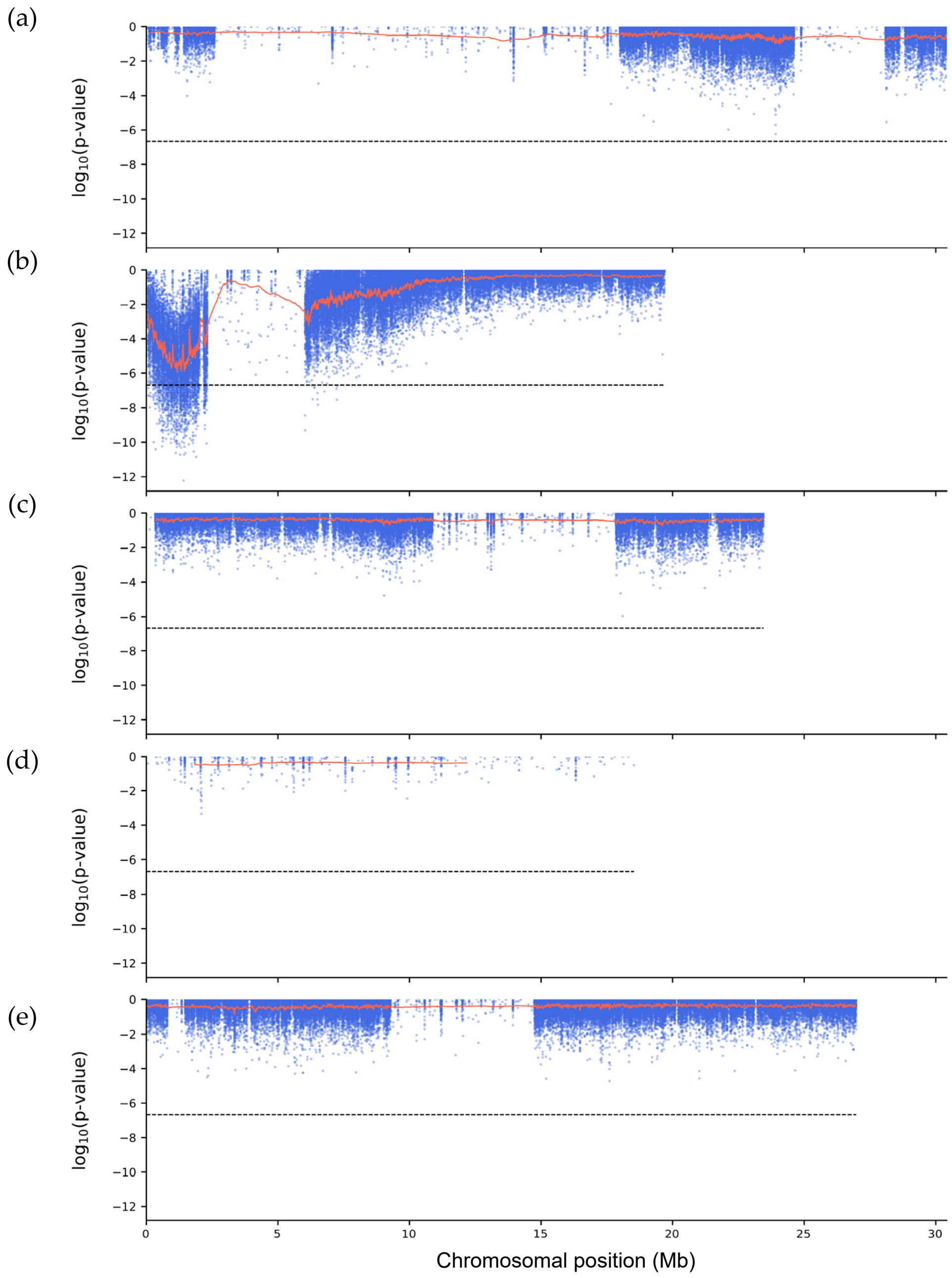

Figure S3. *p*-values of two-tailed Fisher's exact tests performed on the same dataset. (a) Chromosome 1. (b) Chromosome 2. (c) Chromosome 3. (d) Chromosome 4. (e) Chromosome 5. Each blue dot indicates the *p*-value of a biallelic SNP segregating in the population, as determined using the data from both pools. The red line indicates the weighted moving average of the *p*-values of 200 adjacent SNPs.

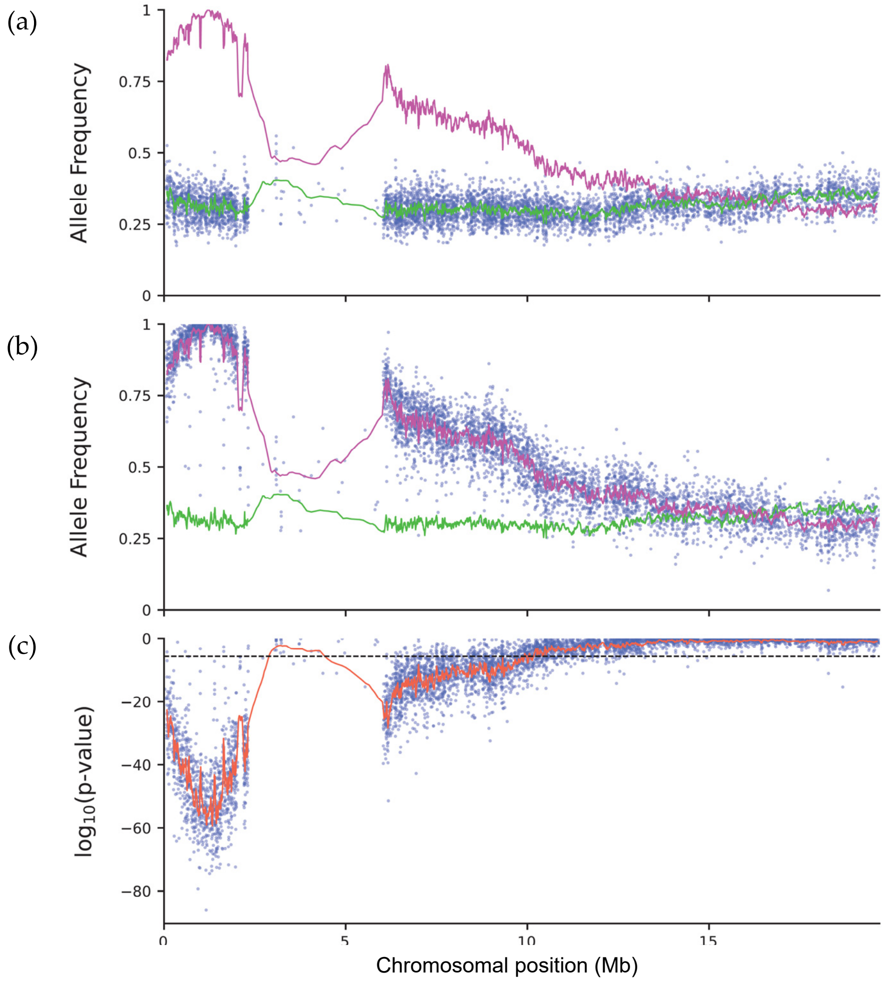

Figure S4. Mapping-by-sequencing of the recessive allele causing the albino seedling phenotype using bins of ten consecutive markers. The plots correspond to genomic regions defined by non-overlapping bins of ten consecutive markers located on chromosome 2. (a) Frequency of haplotypes inherited from the mutant parent in a pool of F_2 seedlings exhibiting the wild-type (photosynthetic) phenotype. (b) Frequency of haplotypes inherited from the mutant parent in a pool of F_2 seedlings exhibiting the mutant (albino) phenotype. (c) *p*-values of two-tailed Fisher's exact tests performed on the same dataset. Each blue dot indicates the allele frequency (a,b) or the *p*-value (c) of a biallelic SNP segregating in the population. The green and pink lines correspond to the moving averages of the values of 20 adjacent bins (i.e., 200 individual markers), determined in the pools of wild-type and mutant individuals. The red line corresponds to the weighted moving average of the *p*-values. The dashed line marks the significance threshold calculated using the Bonferroni correction, considering that n= 23,781 genomic intervals (bins of up to 10 consecutive SNPs) have been tested.

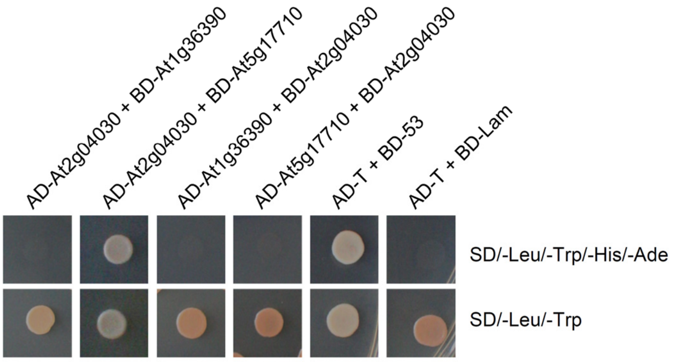

Figure S5. Pairwise yeast two-hybrid assays involving At2g04030 and two GrpE homologs, At5g17710 and At1g36390. AD: constructs containing the activation domain of Gal4; BD: constructs containing the DNA binding domain of Gal4. The interaction between pGBKT7-53 (Gal4 DNA-BD fused with murine p53, indicated as BD-53) and pGADT7-T (Gal4 AD fused with the SV40 large T-antigen, indicated as AD-T) was included as a positive control. The interaction pGADT7-T and pGBKT7-Lam (Gal4 BD fused with Lamin) was included as a negative control.
